# Contribution of Mitochondrial Ion Channels to Chemo-Resistance in Cancer Cells

**DOI:** 10.3390/cancers11060761

**Published:** 2019-05-31

**Authors:** Roberta Peruzzo, Ildiko Szabo

**Affiliations:** 1 Department of Biology, University of Padova, 35131 Padova, Italy; roberta.peruzzo@studenti.unipd.it; 2 CNR Institute of Neuroscience, 35131 Padova, Italy

**Keywords:** mitochondrial ion channels, permeabilization and cytochrome c release, resistance to apoptosis

## Abstract

Mitochondrial ion channels are emerging oncological targets, as modulation of these ion-transporting proteins may impact on mitochondrial membrane potential, efficiency of oxidative phosphorylation and reactive oxygen production. In turn, these factors affect the release of cytochrome c, which is the point of no return during mitochondrial apoptosis. Many of the currently used chemotherapeutics induce programmed cell death causing damage to DNA and subsequent activation of p53-dependent pathways that finally leads to cytochrome c release from the mitochondrial inter-membrane space. The view is emerging, as summarized in the present review, that ion channels located in this organelle may account in several cases for the resistance that cancer cells can develop against classical chemotherapeutics, by preventing drug-induced apoptosis. Thus, pharmacological modulation of these channel activities might be beneficial to fight chemo-resistance of different types of cancer cells.

## 1. Introduction

**Figure 1:**
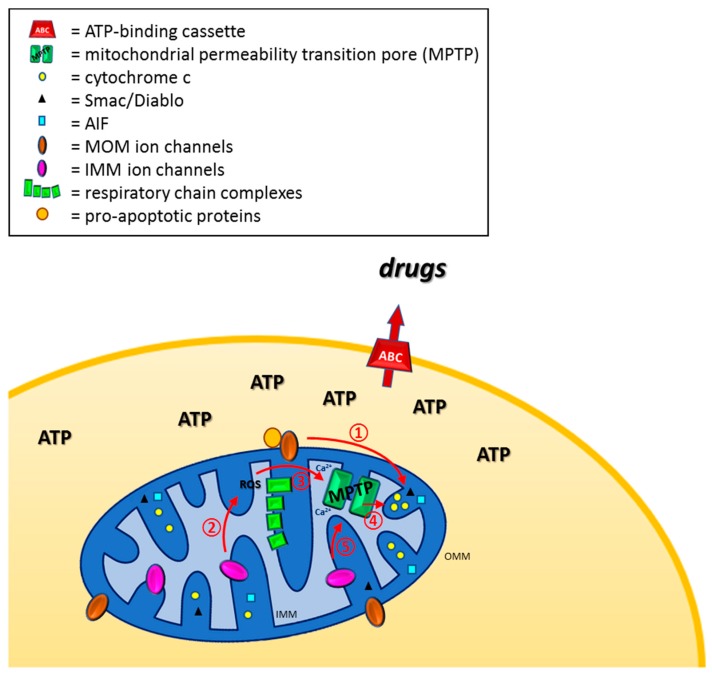
Possible contribution of mitochondrial ion channels to counteracting chemo-resistance. Ion channels and pore-forming proteins of the MOM may directly allow release of cytochrome c and pro-apoptotic proteins from the intermembrane space **①**. Channels of the IMM may: 1) decrease efficiency of oxidative phosphorylation (respiratory chain complexes are depicted as green rectangles) **②**, thereby reducing ATP production that is necessary for the function of ABC type multidrug resistance pumps at the plasma membrane (depicted as ABC); 2) by modulating oxidative phosphorylation efficiency **②**, IMM channels may lead to increased ROS release that in turn triggers opening of MPTP **③** and subsequent release of cytochrome c and other pro-apoptotic factors **④**; 3) by modulating membrane potential of IMM, different IMM channels may trigger MPTP opening **⑤**. See text for further details.

Apoptosis-resistance is of the key hallmarks of cancer cells [1]. Defective regulation of apoptosis importantly contributes to tumorigenesis and cancer progression leading to accumulation of pathologic cells. Mitochondria are central organelles for apoptosis and, in general, for regulated cell death in different organisms [2]. Release of pro-apoptogenic factors, such as cytochrome c, Second Mitochondria-derived Activator of Caspases/ Direct IAP-Binding protein with Low pI (SMAC/Diablo) and apoptosis-inducing factor (AIF) from the mitochondrial inter-membrane space (delimited by the two mitochondrial membranes) represent the point of no return of the intrinsic mitochondrial programmed cell death signaling pathway. Mitochondria may contribute in two major ways to resistance towards chemotherapy: i) by producing ATP, that allows the function of ATP-binding cassette family members, such as multidrug resistance (MDR) proteins that actively extrude xenobiotics (chemotherapeutics) from malignant cells [3,4]; ii) by defective outer membrane permeabilization (MOMP) and/or impaired opening of the mitochondrial permeability transition pore (MPTP) that may prevent release of pro-apoptotic factors, thereby leading to resistance to apoptosis-inducing agents. Beside apoptosis, MPTP is involved also in the mitochondrial permeability transition (MPT) dependent necrosis [5] (Figure 1).

Ion channels of both the outer and inner mitochondrial membrane (MOM and IMM, respectively) might impact a priori on both processes. MOM channels participate in MOMP, while IMM channels fine-tune changes in membrane potential and thereby influence reactive oxygen (ROS) production and efficiency of the respiratory chain [6,7]. ROS in turn may activate MPTP [8] or the caspase-independent ROS-triggered parthanatos (poly (ADP-ribose) polymerase-1 dependent cell death) [5]. In addition, MPTP can be also triggered by Ca^2+^ overload in the mitochondrial matrix or by IMM depolarization and by several other factors (for example oxidative stress, for reviews see [9,10]). Regarding the connection between mitochondrial ion channels, ATP production and MDR function, available information is more limited.

**Figure 2:**
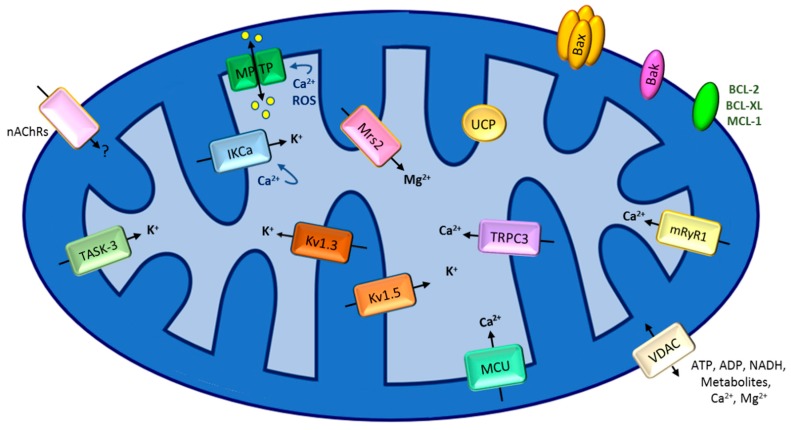
Mitochondrial ion channels and pores involved in apoptosis and chemo-resistance. The figure summarizes the channels/pores treated in this review, i.e., those linked to chemo-resistance. See text for details. The negative membrane potential across IMM (approximately −180 mV) represents considerable driving force for cation entry into the matrix. The nature of the ions transported via the mitochondrial nicotinic acetylcholine receptor has not been defined. UCP mediates the transfer of protons down the electrochemical gradient.

In the present review we summarize our current knowledge regarding the contribution of different classical and of some peculiar ion channels of both MOM and IMM to the modulation of MOMP and MPT, and to other forms of chemo-resistance. Emphasis will be given mainly to recent advances and from the point of view of the channel activities, as MOMP and MPT activation cover a vast literature. Figure 2 summarizes the proteins displaying channel activities that are discussed in the present review. In general, classical MOM ion channels include isoforms of the mitochondrial porin, the mitochondrial form of nicotinic acetylcholine receptor (see below) and an inwardly rectifier potassium-selective channel [11], while IMM channels comprise the calcium uniporter MCU (see below), the magnesium-transporting channel Mrs2 and various K^+^ channels (Big conductance potassium channel (BKCa) [12], Intermediate-conductance K^+^ channel (IKCa) [13], Small conductance K^+^ channel (SKCa) [14], voltage-gated shaker type K^+^ channels Kv1.3 [15], Kv1.5 [16] and Kv7.4 [17], the ATP-dependent potassium channel (mitoKATP) [18], two-pore potassium channel TASK-3 (TWIK-related acid-sensitive K+ channel) [19] (for recent reviews see e.g., [6,7])). In addition, the Inner Membrane Anion Channel (IMAC), the uncoupler proteins (UCPs) and the mitochondrial permeability transition pore (MPTP) (see below) were shown to mediate ion transport in the IMM (for review see e.g., [6]). During the last decades, a considerable number of these distinct channels are being investigated in the context of cancer in addition to the pore-forming BCL-2 family members.

## 2. Defective Mitochondrial Outer Membrane Permeabilization as a Cause of Chemo-Resistance

### 2.1. The Role of Pore-Forming Pro-Apoptotic MOM Proteins of the BCL-2 Family in MOMP

Two extensively studied crucial players of MOMP are two pro-apoptotic BCL-2 family members, namely BAK and BAX. While BAK is resident in the MOM, BAX migrates to the MOM upon various intrinsic apoptotic signals such as for example cytotoxic stress, DNA damage and p53 activation (for reviews see e.g., [20,21]) or upon extrinsic signals transduced to BAX via tBID, a truncated form of BH3-only protein BH3-interacting domain death agonist (BID). Unfortunately, the tumor suppressor p53 is mutated in a considerable number of cancer patients, therefore BAX migration to mitochondria and p53-linked activation of downstream caspases (proteases responsible for the effective, controlled “dismantling” of the cells) is impaired. In addition, BAX expression is very often downregulated in many types of cancer. These two factors, namely p53 mutation and BAX down-expression, prevent BAX-induced MOMP (e.g., [22]) and crucially contribute to drug resistance. Excellent, recent reviews describing the mode of action of BAX/BAK and giving subtle details are available [23,24]. Briefly, BAX activation is a multi-step process characterized by hetero-and homotypic interactions resulting in MOMP that requires the pore-formation by some pro-apoptotic proteins, and possibly other components [25]. The currently accredited view is that BAX oligomers form small pores in the MOM that can initially release smaller intermembrane space (IMS) proteins (such as cytochrome c (13 kDa)) and following further activation, oligomers form leading to pore expansion and the release of larger IMS proteins, such as for example SMAC (54 kDa dimer). The model of this flexible-sized pore formation by BAX reminds the pores formed by the Twin-Arginine Targeting (TAT) system components that, similarly to BAX pores, allow the translocation of fully folded proteins across the thylakoid membrane [26]. In the case of TAT, it has been proposed that an oligomer (comprising 4 to 9 subunits) elicits a severe, destabilizing distortion at the level of intermolecular contacts of the transmembrane helices, leading to local bilayer rupture [27].

The mechanism of pore formation by BAX in the MOM instead has been debated for long time. The intrinsic ability of BAX to form pores/channels has been demonstrated for the first time by incorporating the recombinant, purified protein into planar lipid bilayers, where it formed pH- and voltage-dependent ion-conducting channel with high conductance [28]. The same team reported that two small molecules, able to inhibit BAX channel activity, were also blocking cytochrome c release and prevented ischemic damage of neurons [29]. In accordance, using the electrophysiological technique patch clamp on mitochondria, the MOM was shown to harbor a mitochondrial apoptosis-induced channel (MAC), whose characteristics are very similar to the channels formed by recombinant BAX. Indeed, a correlation between the quantity of BAX molecules and MAC activity suggested that BAX is an essential constituent of MAC [30,31]. Based on cysteine accessibility assay, BAX was shown to insert into the MOM in apoptotic cells via α helices 5, 6 and tail-anchoring helix 9 and to subsequently oligomerize to allow MOM permeabilization [32]. According to a more recent model, dimerization precedes oligomerization and helices α5 and α6 are only partially inserted into the lipid bilayer creating an aromatic planar surface on the membrane, while other helices are embedded into the bilayer [33]. In this scenario, BAX monomers would dimerize and then interact with each other forming a so-called toroid pore and being stabilized by α9–α9 interactions between dimers [24,25]. Interestingly, a BAX point mutation (T182A) exactly in the C-terminal α9 constitutively localizes the protein to mitochondria [34], while a BAX mutant (K128E) harboring the mutation of a highly conserved lysine amino acid residue located between α5 and α6 do not trigger apoptotic downstream signaling anymore and leads to resistance towards multiple apoptosis-inducing agents [35]. The use of such mutants could help elucidation of the molecular details leading to pore formation and the relation of pore formation to cytochrome c release. Evidence for pore formation by BAX has been obtained, in addition to electrophysiology, also by atomic force microscopy of lipid nanodiscs containing BAX [36]. Super-resolution microscopy of GFP-tagged BAX revealed the presence of BAX oligomers with different sizes [37], in accordance with the above-mentioned flexible pore hypothesis. For BAK itself, that similarly to other MOM components might hetero-oligomerize with BAX aiding pore formation, pore formation was not evident [38]. Interestingly, BAX seems to actively permeabilize via pore formation not only the MOM but also the lysosomes, a process of proposed pathophysiological relevance during e.g., Parkinson disease [39].

**Figure 3:**
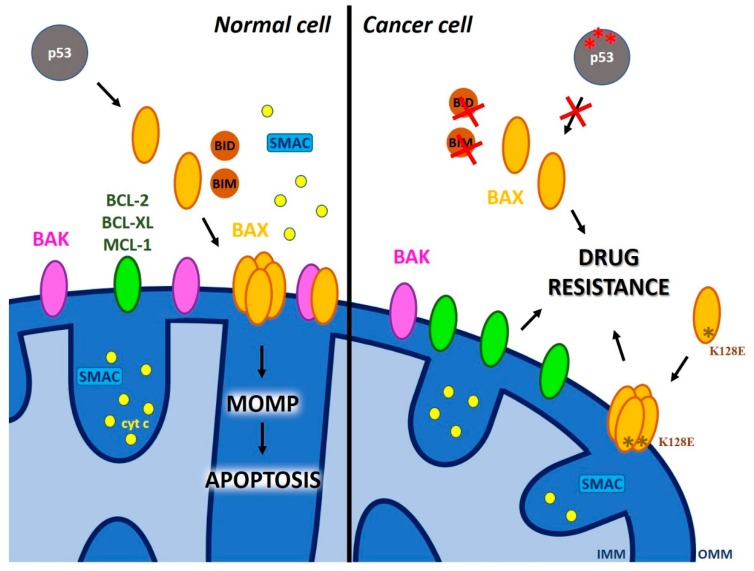
Possible ways of drug resistance due to defective MOM permeabilization (MOMP). In contrast to normal cells where DNA damage leads to p53 activation that in turn triggers migration of BAX to mitochondria and subsequent cytochrome c release leading to apoptosis, in chemo-resistant cells the following events might prevent MOM permeabilization: 1) mutation of p53; 2) mutation of BAX; 3) down-regulation of BAX expression; 4) overexpression of BCL-2 family anti-apoptotic proteins..

Even though BAX oligomerization-triggered channel (pore) formation in the MOM is certainly instrumental for MOMP, the emerging view is that the control of apoptosis and MOMP by BAX/BAK is modulated by additional cellular components [25]. In fact, assembly of BAX oligomers in cells lacking a component of the mitochondrial fission machinery [40], Drp1, was not sufficient to mediate cytochrome c release [37]. Similarly, the observation that both BAX and the BAX K128E mutants are able to form channels in planar lipid bilayer experiments but the latter does not induce cytochrome c release [35], supports the view that additional interactions of BAX are required for pro-apoptotic protein release. Extensive literature deals also with the regulation of BAX/BAK via posttranslational modifications and via their association with various BCL-2 family proteins: i) antiapoptotic proteins (BCL-2, BCLX-L, MCL-1) that counteract BAX pore formation by sequestration; ii) BH3-only activators BID and BIM that promote BAX insertion into MOM. There are also sensitizer proteins, such as BAD and NOXA, that do not directly interact with BAX/BAK but remove the inhibitory effect of anti-apoptotic proteins by sequestration (for recent review see [24]). All these binding interactions are reversible and depend on the equilibrium among the above players (Figure 3). This equilibrium is affected by the expression level of the different BCL-2 proteins—indeed, one of the most often occurring chemo-resistance mechanism is ascribed to the overexpression of anti-apoptotic proteins in several types of cancer, for example in hematologic malignancies [41,42]. Due to intensive exploration in this direction, new molecules targeting anti-apoptotic BCL-2 proteins and affecting this equilibrium, successfully entered clinical trials (e.g., venetoclax [43]). The equilibrium is also affected by affinities of the partners, that may change via modification of the local lipid environment and/or interactions with other molecules (e.g., 14-3-3 proteins [44]). Thus, the final outcome, i.e., formation of large BAX pores allowing MOMP thus likely depends on a plethora of different factors in addition to the ability of BAX itself to form channels. However, direct evidence that lack of pore formation by BAX accounts for chemo-resistance in cells of patients has not been obtained so far, to our knowledge. Once identified, a channel-dead single point mutant of BAX could be expressed to investigate the channel-formation dependent chemo-resistance mechanisms. Alternatively, differences in the channel-forming properties of the N-terminal located P13A BAX mutant could be studied, since WT and mutant BAX display the same subcellular distribution in both healthy and apoptotic cells, but the mutant protein induces a more rapid mitochondrial permeabilization and staurosporine-induced death than the WT protein [45]. No differences were detected concerning membrane insertion and oligomerization between WT and mutant BAX or their interaction with anti-apoptotic proteins or tBID [45], suggesting that the mutation might have introduced a conformational change that accelerates/stabilizes pore formation. This idea could be tested using super-resolution microscopy or AFM, possibly in native membranes.

### 2.2. Dual Role of Voltage-Dependent Anion Channels (VDAC) in Chemo-Resistance

**Figure 4:**
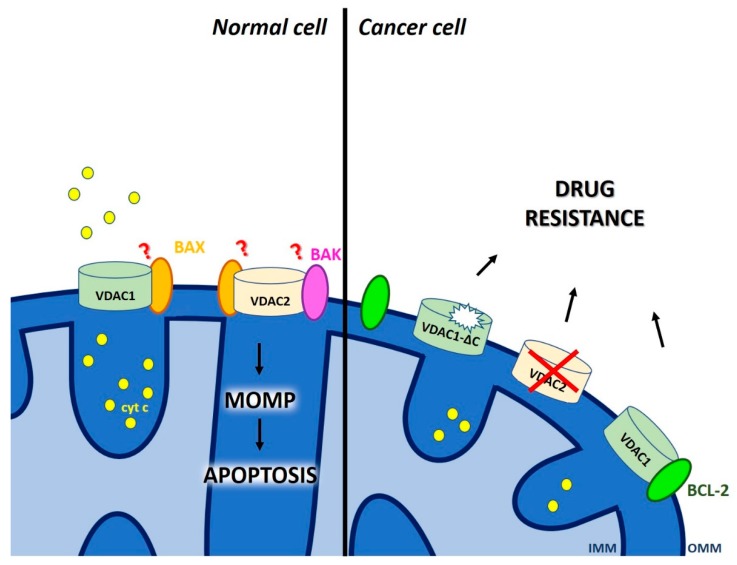
The role of voltage-dependent anion channels in outer membrane permeabilization and in development of chemo-resistance. In healthy cells, VDAC1 oligomerization may lead to MOMP either by homo-oligomerization or by interaction with BAX. Interaction of BAK and BAX with VDAC2 isoform also contributes to MOMP. Instead, expression of a truncated form of VDAC1, association of VDAC1 with anti-apoptotic BCL-2 protein or downregulation of VDAC2 expression contribute to chemo-resistance in cancer cells. See text for further details.

A further class of players in MOMP that reside in the MOM are porins. Mitochondrial porins are also called voltage-dependent anion channels based on their characteristic biophysical properties (opening of the channel with maximal conductance occurs at 0 mV transmembrane potential while higher voltages induce partial closure). The family includes three isoforms, VDAC1, VDAC2 and VDAC3 [46], with VDAC1 and VDAC2 being involved in MOMP (for reviews see e.g., [47,48,49,50]). As the major channel for small hydrophilic molecules in the MOM, VDAC1 mediates flux of metabolites (e.g., ATP, ROS), of ions (e.g., Ca^2+^) and of water across the membrane in physiological conditions, therefore crucially contributing to metabolic pathways and calcium signaling. Given the high conductance of porins, the MOM has long been viewed as a molecular sieve allowing the flux of several molecules without the need of specific transport systems. However, this view has recently been changed, at least in yeast, following the discovery of several new, solute-specific channels in the MOM [51,52,53]. Independently of the exact nature of the molecules crossing through VDAC1, this channel received much attention in the context of tumorigenesis as well as of apoptotic signaling (Figure 4).

VDAC1 is overexpressed in several types of cancer cells [48] and gives a selective advantage to these cells by allowing direct tunneling of ATP (produced in the mitochondrial matrix and exported to the inter-membrane space via the adenine nucleotide carrier) to the first enzyme of the glycolytic pathway, hexokinase. This enzyme is equally overexpressed in many cancer cells and its function contributes to the maintenance of the Warburg effect, as it catalyzes phosphorylation of glucose that enters cancer cells at high rate (e.g., [54,55,56]). The importance of VDAC1 in the context of apoptosis versus survival is illustrated by the findings that VDAC1 expression is linked to chemo-resistance in patients: a truncated but still channel-forming, active VDAC1-ΔC [57] was detected in tumor tissues of late-stage and chemotherapy-resistant lung adenocarcinoma patients [58,59]. The findings indicate that under hypoxic conditions the hypoxia-inducible factor-1 (HIF-1) confers selective protection from apoptosis via induction of VDAC1-ΔC, that allows maintenance of ATP level and cell survival. In another case, transcript analysis from dexamethasone resistant childhood acute lymphoblastic leukemia (ALL) patients revealed a significantly lower expression of VDAC1 with respect to control samples [60]. Thus, VDAC1 might serve as potential prognostic and chemotherapy-response biomarker in childhood ALL. In contrast, silencing VDAC1 expression was found to inhibit cancer cell growth and trigger metabolic rewiring in mouse xenograft models of human glioblastoma (U-87MG), lung cancer (A549), and triple negative breast cancer (MDA-MB-231) [61].

These latter observations might seem contradictory, however the multiple role played by VDAC in cancer cells has to be taken into account when trying to interpret the different results. On one hand, VDAC1 can oligomerize in apoptotic cells, as revealed by chemical cross-linking or by Bioluminescence Resonance Energy Transfer (BRET) assays [62], and in such state, it might be implicated in cytochrome c release [63]. Recently, mutation of VDAC1 E73 to either alanine or glutamine has been shown to reduce dimer formation, which was proposed to play a significant role in mitochondrial metabolic regulation when cytosolic acidification occurs and in cytochrome c release [64]. Another study determined high-resolution crystal structures of oligomeric human VDAC1 and proposed a heptameric structure, which however does not highlight a possible cytochrome c passage pathway [65]. Thus, the exact way how VDAC1 alone could permit cytochrome c release is not fully elucidated.

In addition to self-oligomerization, VDAC1 has also been shown to interact with BAX and proposed to participate in the formation of a pore with sufficient size for pro-apoptotic protein release from IMS [66,67]. Such interaction might be facultative however, as a cyathane-type diterpenoid, that is efficient even in vivo, is able to induce apoptosis in BAX/BAK-deficient cells (by promoting oligomerization of VDAC1), but not when VDAC1 is depleted [68]. Another VDAC isoform, VDAC2 is also able to interact with BAK [69] as well as BAX (e.g., [70,71]). VDAC2 was indeed identified from an unbiased genome-wide CRISPR/Cas9 screen, as a crucial protein for BAX (but not BAK) function. Deletion of VDAC2 resulted in impairment of killing of tumor cells by anti-cancer agents and the ability to suppress tumor formation, similarly to the loss of BAX [72]. On the other hand, high transcript levels of VDAC2 were found to be associated with increased levels of tumor recurrence and resistance to hormonal therapy in high risk breast cancer patients [73].

VDAC1 was also demonstrated to serve as anchoring site for BCL-2 and BCL-XL anti-apoptotic proteins with BCL-2 able to decrease VDAC1 channel conductance [74]. In the same work, the authors also defined the VDAC1 amino acid residues that are important for interaction with BCL-2. Expression of peptides corresponding to the VDAC1 N-terminal region that is mediating interaction with BCL-2, prevented protection against staurosporine-induced apoptotic cell death in BCL-2 overexpressing cells, suggesting that interfering with the binding of BCL-2 to MOM by using these VDAC1-based peptides may potentiate the efficacy of conventional chemotherapeutic agents. In the last years, this hypothesis received confirmation in different systems and preclinical models [75] and the usefulness of these peptides, able to detach hexokinase II as well as BCL-XL in addition to BCL-2, is emerging even in vivo [76,77]. Since VDAC is also a pharmacologically targetable channel [78,79], any information arising from these recent studies might be rapidly exploited, although specificity of action on VDAC1 has to be assessed in order to avoid side effects. To our knowledge, no VDAC isoform specific modulators of channel activity are available, and all three members form channels, although with different properties [80,81]. In summary, both VDAC1 and VDAC2 are important players in chemo-resistance.

### 2.3. Other Proteic Channels and Lipids of The MOM that Modulate MOMP

**Figure 5:**
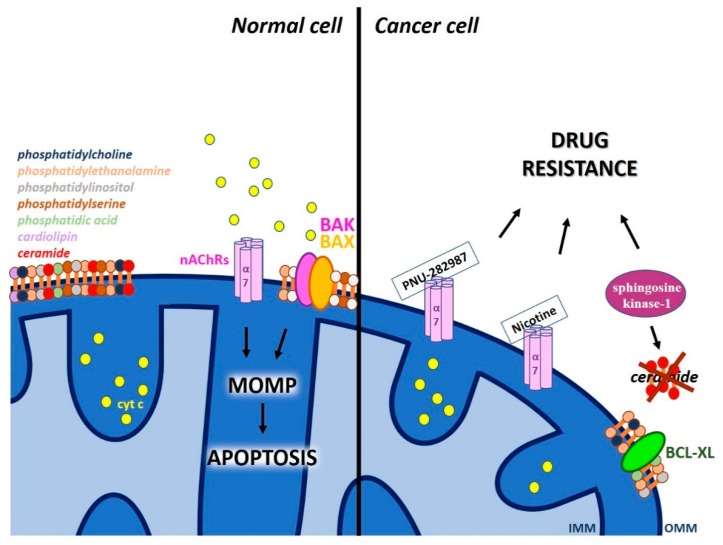
Participation of MOM-located nicotinic acetylcholine receptor and of lipid molecules in MOMP. α7 nAChRs and ceramide channels were linked to MOMP. Agonists of α7 nAChRs downregulate the VDAC-mediated Ca^2+^ transport and thus dampen opening of the permeability transition pore (MPTP), leading to chemo-resistance. BAX can favor, while BCL-XL dampen channel/pore formation by ceramide that would allow release of cytochrome c. See text for further details. Different lipids are shown with different colors (phosphatidylcholine (dark blue), phosphatidylethanolamine (light orange), phosphatidylinositol (light gray), phosphatidylserine (brown), phosphatidic acid (light green), cardiolipin (light violet), ceramide (red)).

Acetylcholine receptors (nAChRs) that are ligand-gated ion channels are found prevalently in the plasma membrane (PM). However, nicotinic α7 AChRs (nAChRs) were shown to be expressed in the MOM and to regulate early proapoptotic events like cytochrome c release [82,83]. Interestingly, the homo-pentameric α7 nAChRs belong to the most ancient branch of this receptor family and are expressed in neurons and non-excitable cells, where they mediate pro-proliferative and anti-inflammatory signaling. Gastric cancer cells, where α7 nAChR expression was knocked-down, showed resistance to 5-fluorouracil (5-FU), a clinically used chemotherapeutic agent [84] but were reportedly more sensitive to docetaxel, paclitaxel and ixabepilone treatment [85,86]. The specific role of mitochondrial nAChRs versus the plasma membrane-located channel was not addressed in these studies. Recently, a protective role of mitochondrial nAChRs in supporting the cell viability during the early phase of liver regeneration was reported [87]. Another study pointing to the importance of mitochondrial nAChRs showed that specific α7 nAChR agonists, such as PNU-282987, impaired intra-mitochondrial Ca^2+^ accumulation at very low, 30 nM concentration, and significantly decreased cytochrome c release stimulated by oxidative stress (Figure 5). Since α7 nAChRs and VDAC were shown to interact in a sandwich Elisa assay, the authors suggested that α7 nAChRs downregulate the VDAC-mediated Ca^2+^ transport and thus dampen the onset of Ca^2+^-induced mitochondrial permeability transition [88]. The α7-containing nAChRs can be activated not only by acetylcholine, but also by choline, which is abundant in the cytosol and can bind to MOM α7 nAChRs. The exact mechanism by which the activated mitochondrial acetylcholine receptor affects VDAC activity has however not been elucidated.

In line with the effect of PNU-282987, nicotine, a specific nAChR agonist was shown to abolish chemotherapy-induced apoptosis and conferred resistance to cell death induced by gemcitabine in pancreatic cancer cells and in pancreatic tumors xenografted into mice [89]. Nicotine can permeate cell membrane and activate mitochondrial nAChRs, that is coupled to inhibition of the MPTP opening [88], thus preventing apoptosis [90] (Figure 5). Thus, the emerging novel concept links activation of plasma membrane nAChRs to growth promotion of cancer cells through activation of various growth factor signaling pathways [91], while activation of mitochondrial-nAChRs would result in inhibition of intrinsic apoptosis through prevention of opening of MPTP. From mechanical point of view, the role of α7 nAChR in chemo-resistance envisions anti-apoptotic activity of nicotine through the activation of the PI3K/AKT pathway, overexpression of survivin, or by induction of BCL-2 through extracellular signal–regulated kinases (ERK) phosphorylation [92]. This proposed chain of events is based on the observation that activated mitochondrial-nAChRs were found to physically associate also with the intra-mitochondrial protein kinases PI3K and Src, resulting in upregulated expression of cyclin D1, activation of ERK1/2 and consequent inhibition of MPTP opening [83]. Thus, MPTP opening seems to be abolished by mitochondrial α7 nAChRs in an indirect way, both by activating ERK1/2 and by reducing Ca^2+^ flux via VDACs.

In addition to the abovementioned proteic channels, lipids of the MOM also seem to play a role in MOMP (Figure 5). In particular, accumulating evidence suggests that MOM lipids promote BAK/BAX activation and pore formation [93]. The MOM contains several ubiquitous lipids, including phosphatidylcholine, phosphatidylethanolamine, phosphatidylinositol, and phosphatidylserine and smaller amounts of phosphatidic acid and possibly cardiolipin (typically found in the IMM). BAX-dependent MOMP was shown to require this latter lipid in vitro [94,95] but not in vivo in yeast [96], so the question of whether cardiolipin is required for MOMP is still unanswered. Sphingolipids are another class of lipids that might alter MOMP through interaction with pro-and/or anti-apoptotic proteins; in particular, multiple sphingolipids cooperate with BAK and BAX to promote MOMP (for summarizing review see [93]). For example, sphingosine-1-phosphate and hexadecenal participate in BAK/BAX activation [97]. The exact step(s) of BAK/BAX activation controlled by these sphingolipid metabolites however remains to be elucidated.

Particular attention can be given to the finding that a specific class of sphingolipids, ceramide themselves can induce large pore formation in the MOM (with diameter up to 10 nm) in a BAX-favored and BCL-XL-inhibited manner (for recent review see [98]). Evidence in intact cells in favor of this hypothesis was obtained: BCL-XL point mutants specifically affecting the interaction between ceramide and the proteic inhibitor BCL-XL were exploited to assess the role of ceramide channels in apoptosis [99]. Interestingly, chemo-sensitive HL-60 acute myeloid leukemia cells are able to generate ceramide upon treatment with drugs, while chemo-resistant cells do not produce ceramide during treatment. Expression of sphingosine kinase-1 resulting in block of ceramide synthesis in chemo-sensitive HL-60 cells resulted in block of apoptosis, that was ascribed to the inhibition of mitochondrial cytochrome c efflux [100]. Measuring the mitochondrial ceramide content in various chemo-resistant primary tumor cells could be useful to further confirm this connection. In any case, the above data are in line with the control of apoptosis by ceramide at the mitochondrial level, however do not prove that indeed the channel formation by ceramide itself is crucial. In this respect, a novel observation regarding the ability of ceramide to specifically bind to VDAC1 and VDAC2 is of relevance: loss of VDAC2 or mutation of its binding site to ceramide rendered the cells resistant to ceramide-induced apoptosis [101].

## 3. Defective Mitochondrial Inner Membrane Permeabilization Leading to Chemo-Resistance

### 3.1. The Mitochondrial Permeability Transition

It has long been known that mitochondria can undergo a Ca^2+^-dependent increase of inner membrane permeability (the permeability transition, PT) causing inner membrane depolarization and interruption of ATP synthesis (see e.g., [9,102]). The PT has been ascribed later on to the opening of a proteic pore, the MPTP, based on the ability of cyclosporin A (CSA) to specifically block the PT [103]. The mitochondrial megachannel (MMC), recorded by direct patch clamping of the IMM, was found to be equally inhibited by CSA [104] and displayed the same pharmacological features of the PT [105,106,107]. This finding further confirmed the proteic pore nature of MPTP/MMC, that requires matrix Ca^2+^ for opening. PTP is favored by IMM depolarization, Ca^2+^ overload in the matrix and by oxidative stress, while it is efficiently inhibited by matrix H^+^, various divalent cations and Mg^2+^/ATP(ADP). Cyclophilin D (CyPD) is instead a protein modulator of the MPTP and acts as a receptor for the high-affinity inhibitor, CSA (e.g., [6]).

MPTP received renewed attention when experimental evidence accumulated showing that long-lasting openings of MPTP may cause matrix swelling and, as a consequence, MOM rupture leading to the release of inter-membrane pro-apoptotic proteins. During cell stress and apoptosis, various pro-apoptotic proteins, including cytochrome c may leave the IMS due to MPTP opening, therefore this process is a critical episode in the chain of events leading to chemotherapy-induced apoptosis. Therefore, the scientific community invested considerable effort to find specific activators of MPTP to be exploited in the context of cancer treatment and of chemo-resistance (for reviews see e.g., [108,109,110]). A number of chemicals and natural substances have been identified (for a list see e.g., [111]), however most studies deal with PTP activation in vitro, and the relatively few studies addressing the in vivo effects of these drugs do not normally investigated possible side effects. One promising example however is hirsutine, extracted from *Uncaria rhynchophylla*, recently shown to exert anti-cancer activity in a lung cancer xenograft mouse model through a signalling cascade leading to GSK3β dephosphorylation and PTP opening [112]. Although the issue of toxicity has not been fully explored, the presented data suggest that hirsutine does not cause additional toxic effects on normal tissues like liver and kidney in vivo. Another useful drug is betulinic acid, a plant-derived triterpenoid that exerts potent anti-cancer effects both in vitro and in vivo, without exerting toxicity towards untransformed cells. This drug induces CSA-sensitive cytochrome c release directly via PTP, even in BAX/BAK-less cells [113]. Betulinic acid is highly efficient against tumor cells of different origin as well as against tumor cells that are resistant to other, classical chemotherapeutic agents. Honokiol from magnolia is another example of a drug able to induce death of a variety of cancer cells by triggering PTP opening and to overcome BCL-2 and BCL-XL-mediated apoptotic resistance. Honokiol was efficient in preclinical models of angiosarcoma [114] and in the case of apoptosis-resistant B-cell chronic lymphocytic leukemia (B-CLL) cells as well as in chemo-resistant multiple myeloma patients’ cells [115]. Importantly, the dose of honokiol that killed cancer cells was not toxic to normal blood cells, suggesting specificity.

The exact way how these and other drugs trigger PTP opening awaits clarification, like the molecular nature of the pore itself. Despite long-lasting research and various hypotheses during the last fifty years (see e.g., [8,9]), the protein(s) constituting the pore is (are) still elusive. In the last years, the hypothesis that PTP originates from specific, Ca^2+^-dependent conformations of the F-ATP synthase [116,117,118] divided the scientific community. To date, evidence based on combination of mutagenesis of F-ATP synthase subunits with single-channel (protein) electrophysiological analysis (see e.g., [119,120,121]) strongly suggests that indeed ATP synthase activity is linked to PTP, although the exact mechanism of pore formation awaits clarification.

### 3.2. Calcium Channels in the Inner Mitochondrial Membrane Linked to Chemo-Resistance

PTP opening can be triggered as mentioned above by Ca^2+^ overload in the matrix. Calcium is imported prevalently via the mitochondrial calcium uniporter (MCU). The molecular identity of MCU was elucidated only less than a decade ago [122,123,124,125,126]. Briefly, MCU complex (MCUC) is currently proven/proposed to be formed in mammals by the pore-forming protein MCU, an MCU paralog (MCUb that acts as dominant-negative pore-forming subunit), the Essential MCU REgulator (EMRE), the regulatory MICU proteins (three isoforms), and the mitochondrial calcium uniport regulator 1 (MCUR1). The best characterized MCUC component in vivo is the EF-hand containing regulatory subunit, MICU1. Patients with loss-of-function mutation of MICU1 display myopathy, cognitive impairment and extrapyramidal movement disorder [127], likely due to an increased agonist-induced mitochondrial Ca^2+^ uptake at low cytosolic Ca^2+^ concentrations and a decreased cytosolic Ca^2+^ signal. Chronic increase of the mitochondrial matrix Ca^2+^ load seems to lead to moderate mitochondrial stress, resulting in fragmentation of the mitochondrial network. MICU1 has also been shown to play a crucial role for tissue repair after injury of liver [128]: in MICU1-deficient hepatocytes Ca^2+^ overload induced PTP opening, a finding that underlines the importance of regulating MCU under stress conditions when the risk of Ca^2+^ overload is elevated.

Beside its physiological role for muscle function, MCUC has been implicated also in the control of tumorigenesis and metastasis. MCU-mediated Ca^2+^ overload might trigger MPTP opening – in accordance, microRNA-mediated (miR-25) downregulation of MCU is associated with resistance to apoptosis in colon and prostate cancers [129]. On the other hand, cells require MCU for cell cycle progression and proliferation and many tumors seem to depend and thrive on a basal level of mitochondrial Ca^2+^ uptake (see e.g., [130,131]). Breast cancer patients’ survival negatively correlated with increased MCU and decreased MICU1 expression [132], suggesting that in particular MICU1 might function as a tumor-suppressor gene. MCU expression was reported to correlate with metastasis and invasiveness of breast cancer also in another work, likely due to its ability to regulate store-operated Ca^2+^ entry (SOCE), that is known to be involved in migration [133]. In an independent study, MCU expression has been related to breast tumour size and lymph node infiltration. Indeed, in a MDA-MB-231 xenograft model, ablation of MCU induced a reduction in tumour growth and metastasis formation [134]. The mechanism proposed to account for slower tumour progression in MCU-lacking cells envisions reduction in mitochondrial ROS production and via HIF-1α and expression of its target genes. In another work, it has been proposed that a small molecule, AG311, shown to retard tumor growth and to reduce lung metastases, might induce breast cancer cell death by activating MCU, although direct proof is missing [135]. More recent work highlights that the receptor-interacting protein kinase 1 (RIPK1) that is upregulated in human colorectal cancer interacts with mitochondrial Ca^2+^ uniporter (MCU) to promote proliferation by increasing mitochondrial Ca^2+^ uptake and energy metabolism [136], suggesting that the RIPK1-MCU pathway is a promising target to treat colorectal cancer. Instead, post-translational modification of MICU1, namely its phosphorylation by a mitochondrial pool of Akt kinase was shown to increase the basal mitochondrial Ca^2+^ level, reactive oxygen species (ROS) production and tumor progression [137]. On the other hand, elevation of mitochondrial calcium level by downregulation of MICU1 and MICU2 has been proposed to occur in pancreatic cancer cells through HINT2, a histidine triad nucleotide-binding (HINT2) protein, whose low expression in patients correlates with poor prognosis and resistance to gemcitabine [138]. Altogether, both MCU and the channel regulator MICU1 may be important targets in the context of chemo-resistant cancers [79,139,140]. Although some new chemical modulators of MCU have recently been identified (for recent reviews see e.g., [79,140]), the affinity of these modulators is considerably lower than that of Ruthenium Red, a rather wide-spectrum inhibitor of MCU. Among the recently synthesized Ruthenium Red analogues [141,142], the membrane permeant Ru265 deserves attention, since it more potent than the widely used Ru360, yet, it preserves selectivity for MCU [142]. The task of finding further high-affinity, yet specific modulators, might be assisted also by structure-activity relationship (SAR) studies based on the recently reported cryo-EM and X-Ray structures of MCU proteins [143,144,145].

As to other possible pathways for calcium, the transient receptor potential cation TRPC3 channel and the mitochondrial ryanodine receptor (mRyR1) may play a role. It is presently unknown whether the mitochondria-located counterparts of these channels or the plasma membrane-located forms contribute to tumor progression.

### 3.3. Inner Membrane Potassium Channels and Chemo-Resistance

A plethora of potassium channels is present in the IMM, many of them having multiple localization within the cells [6,7,146,147]. Many of these channels are highly overexpressed in cancer cells/tissues, giving them a proliferative advantage [148,149]. At the same time, these channels also contribute to apoptosis resistance. For example, the two-pore leak channel TASK-3 is largely overexpressed in almost half of breast tumor cases [150] and seem to promote tumor formation and to confer resistance to hypoxia, at least in vitro [151]. The intermediate conductance calcium-dependent potassium channel (IKCa called also KCa3.1) is expressed in almost all migrating cells and controls proliferation in chronic lymphocytic leukemia (B-CLL), in lung cancer human breast cancer and in hepatocellular carcinoma (for review see [152]). The voltage-gated shaker-type potassium channels Kv1.3 and/or Kv1.5 are overexpressed in various primary cancer cells (e.g., B-CLL) and tissues as well as in cancer cell lines [149,153,154] and a negative correlation between Kv1.3 expression and sensitivity to cisplatin and ceramide was observed, indicating that with decreasing expression of Kv1.3, the resistance of the tumour cells against the cytotoxic drugs increases [154]. Other potassium channels, such as small-and big conductance calcium dependent K^+^ channels, Kv7.4 and the ATP-dependent K^+^ channel instead play a crucial role in defense of the cells against oxidative stress and their pharmacological activation has been reported to exert protective effects against ischemia (for recent review see e.g., [155]).

**Figure 6:**
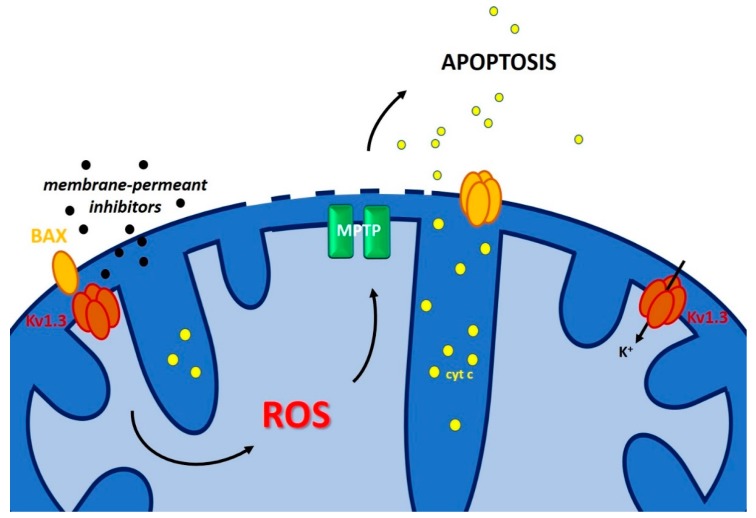
Direct pharmacological targeting of a mitochondrial potassium channel triggers cytochrome c release in cancer cells. Kv1.3 inhibition in the IMM by either BAX or membrane-permeant specific Kv1.3 inhibitors leads to IMM hyperpolarization that in turn triggers ROS release and subsequent ROS-induced MPTP opening. This even in turn results in swelling of mitochondria, loss of mitochondrial integrity and release of cytochrome c, allowing cells to undergo apoptosis.

As mentioned above, TASK-3, IKCa and Kv1.3/Kv1.5 were shown to contribute to apoptosis resistance. However, in many cases no information is available about whether the plasma membrane-located or the intracellular forms of the channels are important for programmed cell death. Information about this point might be obtained by comparing the effects of channel modulators that cannot permeate across the plasma membrane (e.g., toxins, small peptides and hydrophilic compounds) with those that instead pass the membrane (e.g., drugs with hydrophobic nature). For example, membrane-permeant inhibitors of Kv1.3 such as clofazimine, Psora-4 and PAP-1 trigger apoptosis, while the inhibitors acting only on the PM-located channel, such as margatoxin and charybdotoxin do not exert such an effect [156], suggesting that intracellular Kv1.3 is crucial for apoptotic signaling. Likewise, inhibition of IKCa and likely of mitochondrial IKCa (mtIKCa) by membrane-permeant inhibitor TRAM-34 was shown to sensitize melanoma cells to vemurafenib (a BRAF inhibitor) by inducing mitochondrial ROS production [157]. mtIKCa is indeed functional in HeLa cells and in HCT116 colon carcinoma [158], is inhibited by TRAM-34 and clotrimazole [13] and was shown to regulate oxidative phosphorylation in pancreatic ductal adenocarcinoma cells [159], but to our knowledge the effect of TRAM-34 in sensitizing melanoma to vemurafenib cannot be ascribed with high confidence to the mtIKCa versus PM IKCa. The situation is different in the case of mtKv1.3: block of depolarizing K^+^ influx via mtKv1.3 by BAX [35,160] or specific membrane-permeant inhibitors [156,160] was shown to cause IMM hyperpolarization, increase of ROS release, PTP activation, swelling, loss of mitochondrial membrane potential (Δψm), loss of cytochrome c and further ROS release (Figure 6), allowing the cancer cells to reach a critical threshold of oxidative stress, as they are characterized by a higher basal ROS level with respect to healthy cells [161].

In vitro experiments on various cancer cell lines and on primary B-CLL cells demonstrated that membrane-permeant inhibitors of Kv1.3 can kill cancer cells independently of p53 status [162,163,164], BCL-2 overexpression [164] and the presence of BAX and BAK [156]. Subsequent chemical modification of one of these specific inhibitors of Kv1.3, namely PAP-1 [165], to obtain mitochondriotropic PAP-1 derivatives (PAPTP and PCARBTP, obtained by conjugation of the positively charged triphenyl-phoshonium ion (for general strategy see e.g., [166])), increased efficacy of these drugs to trigger cell death and provided evidence that indeed the mitochondria-located Kv1.3 is the channel that is important in the context of apoptosis. In vivo experiments in melanoma and pancreatic ductal adenocarcinoma orthotopic models corroborated the effectiveness of these new mtKv1.3 inhibitors as apoptosis inducers and showed their selective action on pathological cells only [163], as evaluated by the lack of side effects in vivo. This selective action depended on the synergy between high Kv1.3 expression and altered basal redox state in cancer cells, since pretreatment of mice with a molecule able to exert ROS scavenging was able to prevent the tumor-reducing effect of PAP-1 derivatives. mtKv1.3 inhibitors were also able to trigger death in primary tumor cells from patients that underwent classical chemotherapy and became treatment resistant (Peruzzo et al., unpublished). In summary, considerable information points to the possibility to exploit IMM Ca^2+^ and K^+^ channels to trigger death of cancer cells, even of those that are resistant to chemotherapies.

### 3.4. Other Channels

Among the other channels present in the IMM, the magnesium transporter Mrs2 and the uncoupling protein UCP2 deserves attention in the context of this review. An up-regulation of Mrs2 has been observed in a multidrug-resistant (MDR) gastric cancer cell line compared to its parental cells by subtractive hybridization, as well as in several types of cancers according to the Oncomine database [150]. Mrs2 expression positively regulated adriamycin resistance of these gastric cells both in vitro and in vivo, suggesting that high expression of Mrs2 may protect against death [167,168].

UCPs are inner mitochondrial membrane proteins that are able to partially dissipate the Δψm by mediating proton transfer down the electrochemical gradient. UCP-2 is overexpressed in numerous tumors, such as breast, ovarian, bladder, esophagus, testis, colorectal, kidney, pancreatic, lung, prostate cancers and leukemia (for review see e.g., [109,169]). UCP2 overexpression prevented the death-inducing effect of chemotherapy, in particular of Gemcitabine in different contexts [170,171,172]. A decrease in cell viability and clonogenicity were induced following inhibition of UCP2 expression by siRNA and application of tamoxifen in breast cancer cells [173], altogether suggesting that UCP2 expression and function might actively contribute to chemo-resistance in different types of tumors.

## 4. Conclusions and Perspectives

In the present review we summarized the currently available information regarding the roles of mitochondrial ion channels and pore-forming proteins in the regulation of apoptosis and in the context of chemo-resistance. While MOM channels directly regulate MOM permeabilization, IMM channels may trigger cytochrome c release mainly be regulating opening of the MPTP. A great advantage of strategies targeting directly inner membrane channels of mitochondria by pharmacological means is that, as exemplified by the case of mtKv1.3, their modulation might lead to the loss of cytochrome c independently of the outer membrane permeabilization. Therefore, overexpression of anti-apoptotic proteins (e.g., BCL-2, BCL-XL), downregulation of pro-apoptotic proteins (e.g., BAX, BAK) and mutations of p53 and of any apoptotic signaling molecule that is upstream of mitochondria should not prevent cytochrome c release induced by drugs acting directly on IMM channels. Given that many of the discussed IMM channels have a differential expression in healthy and in cancer cells, their modulation, if leading to cytochrome c release, might add a layer of specificity to cancer cell apoptosis induction by a mitochondrial pathway. Table 1 summarizes the effect of drugs acting on mitochondrial channels/pores in the context of cell death/chemo-resistance. In summary, treatment of chemo-resistant cancer cells with mitochondrial channel/pore modulators that trigger a specific series of events leading to apoptosis might become an option of choice in the near future.

## Figures and Tables

**Table 1:** List of ion channels as drug targets against chemo-resistance. The direct effect of AG311 on MCU has not been proven yet.

Ion Channel/Pore	Channel Localization Within Mitochondria	Drug Affecting Channel/Pore Activity	Effect on Cell Death/ Chemo-Resistance	References
BCL-2 anti-apoptotic protein	MOM	Venetoclax (ABT-199)	Kills cancer cells by blocking anti-apoptotic activity of BCL-2	[43]
VDAC1	MOM	cyathane-type diterpenoid	Kills cancer cells even in the absence of BAX/BAK	[68]
VDAC1	MOM	VDAC1-based peptides	Detaches hexokinase II and BCL-XL/BCL-2 from VDAC and potentiates the effect of chemotherapeutics	[76,77]
α7 nAChR	MOM	PNU-282987	Decreases cytochrome c release stimulated by oxidative stress	[88]
α7 nAChR	MOM	nicotine	Confers resistance to cell death induced by gemcitabine	[89]
MPTP	IMM	Hirsutine, betulinic acid, honokiol	Activates PTP and counteracts BCL-2/BCL-XL-mediated apoptosis resistance	[112] [113] [114] [115]
MCU	IMM	Ru265	Inhibits MCU—prevents hypoxia-induced injury (not tested on tumor cells)	[142]
MCU	IMM	AG311	Reduces metastasis	[135]
IKCa	IMM	TRAM-34	Sensitizes melanoma cells to vemurafenib	[157]
mtKv1.3	IMM	PAPTP, PCARBTP	Kills various cancer cells independently of p53 mutation and BAX/BCL-2 expression	[163]
